# *Notes from the Field:* Tularemia Associated with Harbor Seal Necropsy — Kitsap County, Washington, October 2023

**DOI:** 10.15585/mmwr.mm73333a3

**Published:** 2024-08-22

**Authors:** Wendy Inouye, Hanna N. Oltean, Michelle McMillan, Hanna Schnitzler, Beth Lipton, JohnAric MoonDance Peterson, Sylvia DuVernois, Kevin Snekvik, Rebecca M. Wolking, Jeannine Petersen, Elizabeth A. Dietrich, Laurel Respicio-Kingry, Gib Morrow

**Affiliations:** ^1^Kitsap Public Health District, Bremerton, Washington; ^2^Washington State Department of Health; ^3^Washington State Public Health Laboratory, Shoreline, Washington; ^4^Washington Animal Disease Diagnostic Laboratory, Washington State University, Pullman, Washington; ^5^Department of Veterinary Microbiology and Pathology, Washington State University, Pullman, Washington; ^6^Division of Vector-Borne Diseases, National Center for Emerging and Zoonotic Infectious Diseases, CDC.

**Summary**

**What is already known about this topic?**

Tularemia is a zoonotic disease caused by *Francisella tularensis*, a bacterium found in several animal species, most frequently occurring in rabbits and rodents.

**What is added by this report?**

In 2023, tularemia occurred in a wildlife volunteer after exposure to a deceased, infected harbor seal, the first known report of tularemia acquired through contact with a marine mammal, and the first detection of *F. tularensis* in a marine mammal.

**What are the implications for public health practice?**

Health care providers, public health investigators, and persons working with marine wildlife need to be aware of the potential risk for tularemia and other zoonotic diseases associated with harbor seal contact and adhere to established safety protocols.

Tularemia is a zoonotic disease caused by the bacterium *Francisella tularensis*, which has been detected in a wide range of animal reservoirs, most frequently lagomorphs (i.e., rabbits and hares) and rodents (*1*,*2*). Infection can occur through multiple routes, including contact with infected animals, bites from infected insects, ingestion of contaminated water, and inhalation of aerosolized bacteria (*1*). Tularemia can exhibit several distinct clinical manifestations, generally corresponding to the route of exposure. With antibiotic therapy, most patients recover completely. In Washington, fewer than 10 human cases are reported annually (*3*).

## Investigation and Outcomes

On October 20, 2023, a previously healthy woman aged 32 years who lived in Kitsap County, Washington was evaluated by a primary care provider for a painful swelling on the left hand. The patient worked as a wildlife biologist for a nonprofit organization and reported nicking a finger with a scalpel on October 3, while performing a necropsy on a harbor seal (*Phoca vitulina*) that had been found deceased along South Puget Sound.* The patient wore personal protective equipment including a surgical gown, laboratory goggles, an N-95 respirator, and surgical gloves during the necropsy; the cut occurred through the glove. Although the wound initially appeared to heal, it became inflamed and painful 2 weeks after the scalpel cut. Around this time, the patient experienced onset of subjective fever and ipsilateral axial lymph node swelling, as well as cough and congestion. The patient was prescribed doxycycline and topical mupirocin on October 20, and fully recovered. Although tularemia was not suspected by the provider at that time, the wound exudate was collected and submitted to a local clinical laboratory where it was cultured and identified as suspected *Francisella* species. On November 3, 2023, the Washington State Public Health Laboratory received the isolate, where it tested positive for *F. tularensis* by bacterial culture, direct fluorescent antibody, and polymerase chain reaction (PCR).

The seal necropsy report documented signs of possible infection of unspecified etiology in thoracic and abdominal organs without substantial wounds or other signs of trauma. Public health authorities partnered with the Washington Department of Fish and Wildlife to submit animal specimens^†^ to the Washington Animal Disease Diagnostic Laboratory for histopathology and *F. tularensis* PCR testing; six specimens^§^ tested positive by PCR, and three^¶^ were forwarded to CDC’s Division of Vector-Borne Diseases for confirmation. Molecular sequencing (six housekeeping genes; 4,107 base pairs) of the lung specimen performed by CDC identified *F. tularensis* type B (ssp. *holarctica*), phylogenetically similar to type B strains previously found in the western United States (*4*). The clinical isolate from the human case was destroyed in accordance with the Tier 1 select agent handling protocol,** with no sequence generated, prohibiting comparison with the sequence obtained from the seal specimen. This finding is the first known detection of *F. tularensis* in a marine mammal.

Public health authorities identified one other wildlife volunteer present during the necropsy. Contact identification and symptom monitoring of this volunteer and of laboratory workers handling the clinical specimen was conducted by the respective local health jurisdictions. No ill persons or additional cases were identified. This activity was reviewed by CDC, deemed not research, and was conducted consistent with applicable federal law and CDC policy.^††^

## Preliminary Conclusions and Actions

Although most tularemia cases acquired in the northwestern United States are associated with environmental exposure or contact with rodents or lagomorphs, marine mammals should be considered as a potential source of infection. Health care providers, public health investigators, and persons working with marine wildlife need to be aware of the potential risk for tularemia and other zoonotic diseases associated with harbor seal contact and should wear appropriate personal protective equipment and adhere to established safety protocols (*5*).

## Footnotes

* The patient’s organization routinely performs necropsies on harbor seals found deceased in the Puget Sound region to determine whether human interaction potentially contributed to wildlife death.

† Animal specimens included lung, mediastinal mass, trachea, kidney, liver, heart, stomach, small intestine, spleen, brain swab, and lymph nodes.

§ Lung, heart, liver, kidney, brain swab, and lymph node specimens.

¶ Lung, lymph node, and brain swab specimens.

**
https://www.selectagents.gov/compliance/guidance/inventory/docs/Inventory_Guidance.pdf

†† 45 C.F.R. part 46, 21 C.F.R. part 56; 42 U.S.C. Sect. 241(d); 5 U.S.C. Sect. 552a; 44 U.S.C. Sect. 3501 et seq.
